# Subliminal attentional bias modification training for itch

**DOI:** 10.3389/fmed.2023.1104641

**Published:** 2023-05-18

**Authors:** Jennifer M. Becker, Dimitri M. L. Van Ryckeghem, Stefaan Van Damme, Geert Crombez, Yalou Schoot, Reinout W. H. J. Wiers, Ralph C. A. Rippe, Antoinette I. M. van Laarhoven

**Affiliations:** ^1^Health, Medical and Neuropsychology, Faculty of Social and Behavioural Science, Institute of Psychology, Leiden University, Leiden, Netherlands; ^2^Department of Experimental-Clinical and Health Psychology, Faculty of Psychology and Educational Sciences, Ghent University, Ghent, Belgium; ^3^Section Experimental Health Psychology, Department of Clinical Psychological Science, Faculty of Psychology and Neuroscience, Maastricht University, Maastricht, Netherlands; ^4^Research Unit INSIDE, Faculty of Humanities and Social Sciences, Institute of Health and Behavior, University of Luxembourg, Esch-sur-Alzette, Luxembourg; ^5^Addiction Development and Psychopathology (ADAPT) Laboratory, Department of Psychology, University of Amsterdam, Amsterdam, Netherlands; ^6^Research Methods and Statistics, Faculty of Social and Behavioral Sciences, Institute of Education and Child Studies, Leiden University, Leiden, Netherlands

**Keywords:** itch, pruritus, attention, cognitive bias, unconscious processing, experimental psychology, psychodermatology

## Abstract

**Introduction:**

Itch is unpleasant and induces the urge to scratch. This is adaptive to remove the itch-inducing stimulus from the skin. Accordingly, itch draws attention to protect our bodily integrity. Recent studies investigated whether attention is preferentially drawn towards its location, i.e., attentional bias (AB), and also whether this bias could be changed in healthy individuals. So far, results are mixed concerning the existance of an attentional bias towards itch stimuli in healthy individuals as well as the impact of modifications. However, available studies have typically focused on conscious processing and might miss preconscious aspects of attention and potential biases at these stages.

**Methods:**

This study included 117 healthy individuals who underwent a subliminal Attentional Bias Modification (ABM)- training for itch based on a dot-probe paradigm with itch- related pictures. Participants were randomly assigned to a training towards itch group, a training away from itch group and a control group. This was done by manipulating the itch-target congruency of the dot-probe task during a training block. Pre- and post-training assessments were regular dot-probe tasks. Exploratorily, also attentional inhibition, cognitive flexibility and itch-related cognitions were assessed. Lastly, participants received an itchy stimulus on the inner forearm before and after the ABM-training to assess potential effects on itch sensitivity.

**Results:**

Results showed no AB towards itch across groups at baseline, i.e., pre-training, but an AB away from itch, hence, avoidance of itch, post-training. Further analyses showed that this effect was driven by an attentional bias away from itch in the control group, while there were no significant effects in the experimental groups. There was no effect on itch sensitivity.

**Conclusion:**

These findings are in line with recent studies on conscious ABM-training for itch and pain that also did not find significant training effects. Therefore, it is suggested that the field of AB might need to reconsider the current assessment of AB. Moreover, AB is probably a dynamic process that is highly dependent on current itch-related goals and relevance of itch in a specific situation. This suggests that processes probably differ in patients with chronic itch and that also ABM-training might work differently in these populations.

**Clinical trial registration:**

https://trialsearch.who.int/Trial2.aspx?TrialID=NTR7561, identifier NTR7561.

## 1. Introduction

Itch is an unpleasant sensation which induces the urge to scratch and can lower individual’s quality of life if it is present for a prolonged time (1–4). Recent studies have highlighted the importance of psychological mechanisms in the experience of itch, such as attention (5–7). Specifically, it has been suggested that the experience of itch is impacted by attentional processing (8–10). Although attention allocation towards itch-related stimuli may be helpful in adapting our behavior to protect bodily integrity, it can also interfere with the execution of other tasks in daily life. This is especially true if itch can no longer be adaptively controlled, e.g., chronic itch; no concrete action allows to alleviate the itch.

Overall, research on attention to itch showed that, in healthy individuals, itch interferes with the execution of other tasks, i.e., itch is distracting (9, 11, 12). Furthermore, it has been researched whether visual itch-related stimuli draw attention towards their location, i.e., an attentional bias towards itch, which resulted in mixed findings so far (9–11). These studies have shown that attention for itch might differ between conscious and preconscious processing stages: while some studies found heightened conscious attention towards itch (9), others could not replicate this finding (10, 11) and a recent finding suggests preconscious avoidance of itch-related stimuli (13). The importance of fast processing of itch is also supported by contagious itch which suggests very fast and maybe unconscious processing of itch-related gestures, e.g., scratching, which then induces itchiness in the observer (14, 15).

A possible intervention for biases for itch-related information is Attentional Bias Modification (ABM) training for itch. These kind of trainings use itch-related stimuli, like words or pictures to manipulate individuals’ attention away from (or towards) these stimuli. As yet, only one study employed an ABM-training for itch in healthy individuals which investigated conscious processing of itch-related visual stimuli (16). This study investigated whether attention could be either trained towards visual itch stimuli or away from these stimuli. Results of this study could, however, not support the effectiveness of an ABM-training, neither by affecting attention directly, nor by influencing individuals’ sensitivity to a light cutaneous itch stimulus on the skin (16).

Nevertheless, there is some evidence that ABM-training for other somatic complaints such as pain can be effective (17–20), although this could not be supported by all studies (21, 22). Interesting to note here is that in most cases there was no direct effect on attentional bias towards pain stimuli after the training but effects on for example pain intensity or tolerance (17–19). This suggests that ABM-training might show effects on symptom perception, for instance itch tolerance or sensitivity, which could be especially valuable for clinical practice. After all, the lack of significant effects on attentional bias measures themselves leaves open questions about the working mechanism of ABM-training.

Because attention is a continuum, including first orienting towards a stimulus, actual selective attention to a stimulus and eventual disengagement (23–25), attention can be biased at different stages of attentional processing (26) which is suggested by the inconsistend findings on attentional bias towards itch so far at different processing stages, e.g., conscious engagement and disengagement vs. preconscious orienting (9–11, 13). However, preconscious ABM-trainings are scarce and actually lacking in itch. To our knowledge, there is only one study which investigated preconscious ABM training. This study used an ABM training for threat-related stimuli in socially anxious individuals (27) which, while not finding an effect on attentional bias, did find a positive effect on anxiety during a stressful task. This finding indicates that training attention away from itch-related information very early in the attention process may prove helpful in reducing negative outcomes.

With the very limited knowledge on preconscious ABM-training and attention towards itch in general, the current study investigated the effect of preconscious ABM-training for itch in healthy individuals in a proof-of-principle approach. More specifically, the effects on attentional measures and on sensitivity to a somatosensory itch stimulus were investigated. Participants were either trained towards or away from visual itch stimuli or received a sham (control) training by means of computerized, single-session ABM-training. We expected an effect on attentional bias post-training compared to pre-training in both training groups, i.e., more attention towards itch in the towards group vs. less attention towards itch in the away group, compared to the control group. In line with this, we expect higher itch sensitivity after the training in the towards group, and lower itch sensitivity in the away group, compared to the control group. In addition, a possible role of general attentional abilities, namely attentional inhibition and cognitive flexibility, as well as on self-reported itch-related cognitions was explored to shed more light on individual differences that might be related to the effectiveness of the ABM-training and could potentially explain mixed-findings in this field.

## 2. Materials and methods

### 2.1. Participants

The study sample consisted of 117 healthy individuals. This sample size was calculated in line with an earlier study with a comparable design (16). Participants were included if aged between 18 and 35 years, fluent in either Dutch or English, and with normal vision (corrected with contact lenses if needed). Participants were excluded if they had a (history) of psychological disorder (e.g., depression or anxiety), had a medical diagnosis (e.g., atopic dermatitis or heart disease), used recreative drugs on a regular basis (e.g., MDMA or cannabis) or suffered from color blindness or dyslexia. All participants gave written informed consent before the experiment. Data collection took place between October 2018 and July 2019. The study was approved by the Psychology Research Ethics Committee of Leiden University (CEP19-0703/376) and registered in the Nederlands Trial Register (Dutch Trial Register; NTR7561).

### 2.2. General procedure

**Figure 1:**
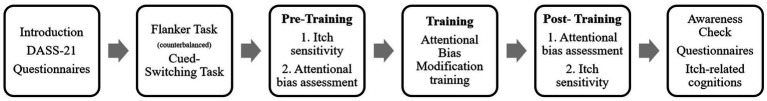
Overview of the general procedure.

Participants were recruited via the Online Research Participation system of the university (SONA Systems Ltd., Tallinn, Estonia) and via advertisement at the faculty. The experiment took place at the Faculty of Social and Behavioral Science of Leiden University and took about 1.5 h. See Figure 1 for an overview. Information about the study was given upon sign-up and repeated at the start of the study, after which participants signed the informed consent form. The procedure started with a short questionnaire about current levels of depression, anxiety and stress and demographic information. Thereafter, two general attention tasks (order counterbalanced) were completed, measuring attentional inhibition and cognitive flexibility. Next, an itchy stimulus was applied to the forearm of the participant to assess their itch sensitivity at baseline (randomized either the dominant or non-dominant arm). The actual subliminal attention bias modification (ABM) training was completely automatized with a pre-training, i.e., baseline- attentional bias block, and a post-training block and the training block in between. Group allocation was based on participant number and the experimenter and the participants were unaware of the corresponding group, i.e., a blinded design. A second itch sensitivity assessment followed by applying the same itch stimulus on the other forearm of the participant (e.g., dominant arm if first application was on the non-dominant arm). Lastly, participants filled out several questionnaires, assessing itch-related cognitions, e.g., catastrophizing and body vigilance. All participants were debriefed and received either monetary reimbursement or course credits for their time investment.

### 2.3. Technical set-up

All computer tasks, including the ABM-training, were programmed with E-Prime 2.0 (Psychology Software Tools, Inc., Sharpsburg, United States) and self-report questionnaires were presented with Qualtrics (Provo, Utah, United States) on an Iiyama HM703UT A Vision Master Pro 413 CRT monitor (17 inch; refresh rate 100 Hz; resolution 1,024 × 768px). Participants used a chin rest to keep a constant distance of 78 cm to the screen. Responses were collected with a Serial Response Box (Psychology Software Tools, Inc., Sharpsburg, United States) with two custom-made buttons for the left and right index fingers. A Tobii Pro X3-120 Eye Tracker (Tobii AB, Danderyd, Sweden) was also installed to measure eye-movements during the ABM-training. Unfortunately, data quality of eye-movement data appeared to be insufficient for further analyses.

### 2.4. Attention tasks

#### 2.4.1. Subliminal attentional bias assessment and training

Attentional bias towards itch was measured with a dot-probe paradigm (9–11). Forty pairs of two pictures were used, one being itch-related and one being neutral (i.e., 20 stimuli presenting neutral skin and 20 presenting a neutral object), validated and used in earlier studies (10, 13). An itch-related picture showed someone scratching their own body. Neutral skin pictures displayed the same body parts, but without a scratching gesture.

Each trial began with a fixation cross (500 ms) followed by a picture pair (20 ms). The picture pair was thereafter masked with corresponding scrambled versions of the same pictures (480 ms). The pictures were presented at the 80 and 20% height position of the screen. Lastly, a target appeared which consisted of two dots, either horizontally or vertically oriented. If the target appeared in the same location as the itch-picture, this was a congruent trial, while if the target appeared in the opposite location, this was an incongruent trial. Participants had to respond to the orientation of the dots by pressing a left button with their left index finger to indicate vertical dots or a right button with their right index finger to indicate horizontal dots or vice versa (counterbalanced). Accuracy and reaction times were assessed as outcome measures. Attentional bias towards itch is inferred if congruent trials have a shorter reaction time (RT) than incongruent trials, while attentional bias away from itch (i.e., avoidance) is inferred if incongruent trials have a shorter RT than congruent trials. The resulting difference score is called the AB-index. The whole ABM-training, including pre- and post-training assessment, took about 30 min to complete.

In line with an earlier study for itch (16), participants were distributed across three groups: one trained towards itch (towards-group), one trained away from itch (away-group) and one control-group (sham training). For each participant, the picture pairs were randomly distributed to the pre-training, training and post-training block.

##### 2.4.1.1. Pre- and post-training attentional bias

For the pre-training, i.e., baseline, assessment of attentional bias towards itch, and the post-training attentional bias towards itch assessment, 10 picture pairs (different picture pairs: for baseline and post-training assessment) were used. All pairs appeared two times: with the itch picture in the upper and lower part of the screen, as a congruent and incongruent trial, and with horizontal and vertical dots, resulting in 160 trials. A break of 10 s was inserted after every 40 trials.

##### 2.4.1.2. Training

For the training, 20 picture pairs (different from baseline and post-training assessment) were presented two times in both locations and with both targets types. The task was manipulated for the towards-group by only consisting of itch-congruent trials and for the away-group by only consisting of itch-incongruent trials. The control-group received evenly distributed congruent and incongruent trials, alike the pre- and the post-training. The whole training block consisted of 320 trials, also interrupted with 10 s breaks after every 40 trials.

##### 2.4.1.3. Awareness check

Awareness of the subliminally presented pictures during the ABM-training, was checked by two subjective awareness questions and an objective awareness check in line with an earlier study (13). Subjective awareness was assessed by directly asking whether participants noticed something special during the task (question 1) and if this was answered with yes, whether they noticed any pictures (question 2). For the objective awareness check, a forced-choice paradigm was used. Participants were presented with 20 picture pairs that consisted of one picture shown during the ABM-training and one new picture from the same validated stimulus set (10). For each pair, they had to indicate which of the two pictures they had seen earlier during the ABM-training. There was no time pressure, but participants were asked to answer as intuitively as possible. Accuracy was measured and if this was at chance level (ca. 50%), the subliminal design was assumed to be successful.

#### 2.4.2. Flanker task

General attentional inhibition, unrelated to itch, was measured with a Flanker paradigm (28, 29) to assess any individual differences in attentional inhibition that might influence an AB towards itch. During each trial within this task, a target number appeared in the middle of the screen, flanked by either two target-identical flanking numbers on each side (i.e., congruent trial) or two different flanking numbers on each side (i.e., incongruent trial). Stimuli were twos and fours, e.g., “22222” or “22422”. Numbers were shown until a response was given, with a maximum of 1,500 ms. After eight practice trials, 120 trials were presented (50% congruent and 50% incongruent) with a short break in the middle. Accuracy and reaction times to respond to the target (middle) number were measured. Attentional inhibition is inferred if incongruent trials have a longer RT than congruent trials, that is, more time is needed to inhibit the incongruent flanking numbers. This is called a Flanker effect (Flanker Index = RT_incongruent_ – RT_congruent_). The Flanker task took about 5 min to complete.

#### 2.4.3. Cued-switching task

General attentional switching, unrelated to itch, was measured with a cued-switching paradigm (28). On each trial of the cued-switching paradigm, a target number between one and nine appeared on the screen. Before the target number appeared, one of two instructions were given for 500 ms: either to indicate by button press whether the target is odd or even (“odd/even”) or whether the target is above or below five (“high/low”). Target numbers were shown until a response was given, with a maximum of 1,500 ms. After 16 practice trials, 200 experimental trials were administered (50% odd/even, 50% high/low) with a short break after 100 trials. Trials could be either repeat-trials (same instruction as preceding trial, 50% of trials) or change-trials (other instruction than preceding trial, 50% of trials). A switching cost is inferred if change trials have longer reaction times than repeat trials, that is, switching from one instructions to another instructions costs time. This is called switching cost (RT_change_ – RT_repeat_). Accuracy and reaction times to respond to the targets was assessed as outcome measure. The cued-switching paradigm took about 10 min to complete.

### 2.5. Itch sensitivity

General itch sensitivity was assessed by applying cowhage spicules (hairs of the tropical *mucuna pruriens* plant) on the inner forearm of the participants. Forty to forty-five spicules were taken with negative grip tweezers (Dumont Tweezers Negative Action Style NS, Electron Microscopy Sciences, Switzerland), counted with the aid of a Bresser microscope Advance ICD 10x-160x (Meade Instruments Europe GmbH & Co. KG, Rhede, Westfalen, Germany). The spicules were applied to a 1.5 cm by 1.5 cm area on the inner forearm, 1 cm above the wrist. The area was demarcated with 1.25 cm surgical tape (3 M Transpore White, St. Paul, MN, United States). The experimenter gently rubbed the spicules, with the index finger, onto the skin for 45 s. Thereafter, participants rated their itch level continuously for 3 min on a digital Visual Analogue Scale (VAS) ranging from zero (“not at all”) to ten (“worst imaginable itch”) on a Lenovo Tab 4 10 Plus (Lenovo Group Limited, Beijing, China). The VAS was displayed with the APK Pure VAS App 1.3 (30). After 3 min, the spicules were removed by rapidly attaching and removing a 2.5 cm surgical tape (3 M Transpore White, St. Paul, MN, United States) to the demarcated area for five times. After another 3 min, participants rated their current itch once orally on a numeric rating scale from zero to ten. If the answer was above one, participants indicated their current level of itch again after another 2 min to make sure that the itch had passed before continuing the session.

### 2.6. Self-report questionnaires

Besides general demographic information and information about in- and exclusion criteria, several questionnaires were administered. The Depression, Anxiety and Stress Scale- short version (DASS-21) (31, 32); the Pain Vigilance and Awareness Questionnaire- adjusted for itch (10, 33, 34); the Experience of Cognitive Intrusion of Pain scale- adjusted for itch to assess cognitive intrusions about itch (10, 35); and the Pain Catastrophizing Scale- adjusted for itch (10, 36). These questionnaires were used to assess emotional distress, vigilance to itch, intrusive cognitions about itch and catastrophizing about itch, respectively. Lastly, one item about disengagement from itch (12) was measured, as well as current level of itch and fatigue with two VAS scales ranging from zero (“not at all”) to ten (“worst imaginable”). These questionnaires were administrered to explore the effect of itch-related cognitions on an AB towards itch.

### 2.7. Statistical analyses

Data of the computer tasks was extracted with E-Prime Data Aid 3.0 (Psychology Software Tools, Inc., Sharpsburg, United States). For the dot-probe pre-training and post-training task the following data was extracted for all experimental trials: reaction times (RT, ms), accuracy, congruency, group and trial number. In addition, mean accuracy levels per participant were extracted for the training itself. For the Flanker task, mean RT, separately for congruent and incongruent trials, and accuracy were extracted for each participant. Likewise, for the cued-switching task mean RT for the change-trials and for the repeat- trials were extracted, as well as mean accuracy. In both tasks, only trials that were responded to correctly and with RT > 150 ms were included for the mean calculations. As explained in Sections 2.4.2 and 2.4.3, respectively, a Flanker index (attentional inhibition) and switching costs (cognitive flexivility) were calculated to use as predictors during statistical analyses. For the questionnaires, data was extracted from Qualtrics (Provo, Utah, United States) and total scores and reliability scores were calculated with SPSS (IBM Statistics for Windows, Armonk, NY, United States). Itch sensitivity data was operationalized as Area Under the Curve (AUC) during the 180 s that were rated on the digital Visual Analogue Scale. AUC was calculated for each participant’s pre- and post-training itch induction.

All subsequent analyses, as described below, were done with R Version 4.0.4 (37) with a significance level of 0.05. Descriptive statistics are given as mean (*M*) and standard deviation (*SD*) if not stated otherwise. Reliability of the dot-probe pre- and post-training was calculated with the package “splithalfr” (38) in line with earlier studies (13, 16).

#### 2.7.1. Manipulation and baseline checks

The objective awareness measure was analyzed with a single proportion test to check if accuracy to detect the picture that was shown during the subliminal pre-training dot-probe task was at chance level (0.5). Subjective awareness (i.e., aware of something and aware of pictures) was investigated with frequency tables.

Baseline between-group differences were checked with bootstrapped (1,000 samples) analyses of variance (ANOVA) with group (control vs. towards vs. away group) as between-subjects effect. This was done for age, the Flanker index, switching costs, self-report questionnaire scores and the pre-training itch-sensitivity AUC score. Gender distribution across groups was assessed with a chi-square test.

#### 2.7.2. Attentional bias pre- and post-training

For the pre- and post-training analyses, only trials with RTs > 150 ms were included. Furthermore, all variables were checked visually for extreme values. For the post-training, only participants who had an accuracy level of at least 0.70 during the training were included (16).

Pre-training attentional bias was analyzed with a mixed-model analysis with RT as dependent variable and random effects for participant and trial number. Model 1 included fixed effects for accuracy, congruency (congruent vs. incongruent) and group (away vs. towards vs. control) as well as the interaction between congruency and group. In Model 2, the Flanker index (and its interaction with congruency), switching costs (and its interaction with congruency) and self-report scores were added as covariates. Post-training attentional bias was analyzed with the same mixed- models (Model 3 and 4, respectively) but added pre-training AB index (RT_congruent_ – RT_incongruent_) as a covariate to control for baseline attentional bias effects. A negative AB index indicates that attention is biased towards itch (see Section 2.4.1).

#### 2.7.3. Itch sensitivity pre- and post-training

Itch sensitivity was analyzed with bootstrapped (1,000 samples) ANOVA on cowhage evoked itch scores (AUC) with group as between-subject effect. Again, pre-training itch scores (AUC) were added as a covariate in the post-training analysis to control for any baseline effects.

## 3. Results

### 3.1. Participants and baseline characteristics

The final sample of 117 participants was mostly female (86% female and 14% male) with a mean age of 21.0 years (SD = 2.3). Table 1 shows descriptive statistics for all self-report questionnaires and the flanker and cued-switching paradigm. As expected, participants showed a significant Flanker index, *t*(231.98) = −4.99, *p* < 0.001, and a significant switching cost, *t*(223.33) = −3.55, *p* < 0.001. Overall, scores on self-reported itch-related cognitions were low to moderate in the current sample with a high dispersion of individual scores. There were no significant differences between all three groups on any background variables (all *p* > 0.05).

**Table 1:** Descriptive statistics (mean (*M*) and standard deviation (SD)) for all background variables.

	Total sample *N* = 117		Control group *N* = 42		Towards group *N* = 38		Away group *N* = 37		*p*-value
	*M* (SD)	Range	*M* (SD)	Range	*M* (SD)	Range	*M* (SD)	Range	
Age^a^	21.0 (2.3)	18–29	21.0 (2.3)	18–26	21.2 (2.6)	18–29	20.8 (2.1)	18–25	0.807
PVAQ-I	41.3 (14.7)	5–74	40.0 (12.4)	9–67	42.7 (15.2)	5–74	41.3 (16.4)	10–74	0.650
PCS-I	23.0 (9.0)	0–45	21.8 (9.0)	0–45	24.0 (8.9)	0–43	23.3 (9.0)	2–43	0.412
ECIP-I	11.1 (9.0)	0–48	10.7 (10.1)	0–48	13.2 (11.4)	0–39	9.9 (11.6)	0–45	0.758
DASS-Depression^b^	7.2 (4.6)	0–19	7.4 (4.2)	0–16	7.3 (4.9)	0–17	6.8 (4.9)	0–19	0.592
DASS-Anxiety^b^	7.1 (4.1)	0–17	7.1 (3.9)	0–17	7.4 (4.3)	0–14	6.7 (4.1)	0–14	0.748
DASS-Stress^b^	9.5 (4.8)	0–18	9.4 (4.0)	0–17	9.7 (5.0)	0–18	9.2 (5.4)	0–17	0.889
Diseng-I	3.7 (1.0)	1–5	3.9 (1.0)	2–5	3.4 (0.9)	1–5	3.7 (1.1)	1–5	0.297
Flanker Index (ms)	46.7 (27.3)	−25.5 to 141.2	44.0 (25.0)	−0.1 to 123.7	50.5 (27.2)	6.49–133.0	44.7 (29.5)	−25.7 to 141.2	0.991
Switching cost (ms)	133.7 (118.4)	−41.5 to 551.9	153.9 (130.6)	−21.1 to 551.9	112.6 (90.0)	−6.9 to 319.1	132.5 (125.5)	−41.5 to 481.8	0.422

*p*-values with bootstrapped residuals are reported to indicate significant group differences due to skewed distributions.

a Total sample *n* = 116; Control group *n* = 41, due to one missing value.

b Total sample *n* = 113; Control group *n* = 38, due to four missing values.

PVAQ-I = Pain Vigilance and Awareness Questionnaire -adjusted for itch (0–80); Cronbach’s alpha = 0.91.

PCS-I = Pain Catastrophizing Scale -adjusted for itch (0–52); Cronbach’s alpha = 0.91.

ECIP-I = Experience of Cognitive Intrusions of Pain Scale -adjusted for itch (0–60); Cronbach’s alpha = 0.97.

DASS = Depression, Anxiety, and Stress Scale- short form (0–21 for each subscale).

Cronbach’s alpha _Depression_ = 0.94; Cronbach’s alpha _Anxiety_ = 0.88; Cronbach’s alpha _Stress_ = 0.91.

Diseng-I = One item on ability to disengagement from itch (1–5).

### 3.2. Pre-training

During the pre-training attentional bias measurement, 3% of the data had to be excluded due to trials with RT < 150 ms, data due to an extreme value of two participants’ switching costs, and data due to one participant’s low accuracy during the task. Reliability analyses showed high reliability for congruent trials, with a mean Spearman-Brown coefficient of 0.97 [Interquartile Range (IQR) = 0.96; 0.97]. Likewise, for incongruent trials, the mean Spearman-Brown coefficient was 0.96 (IQR = 0.96; 0.97). AB index reliability had a mean Spearman-Brown coefficient of 0.43 (IQR = 0.36; 0.52).

**Figure 2:**
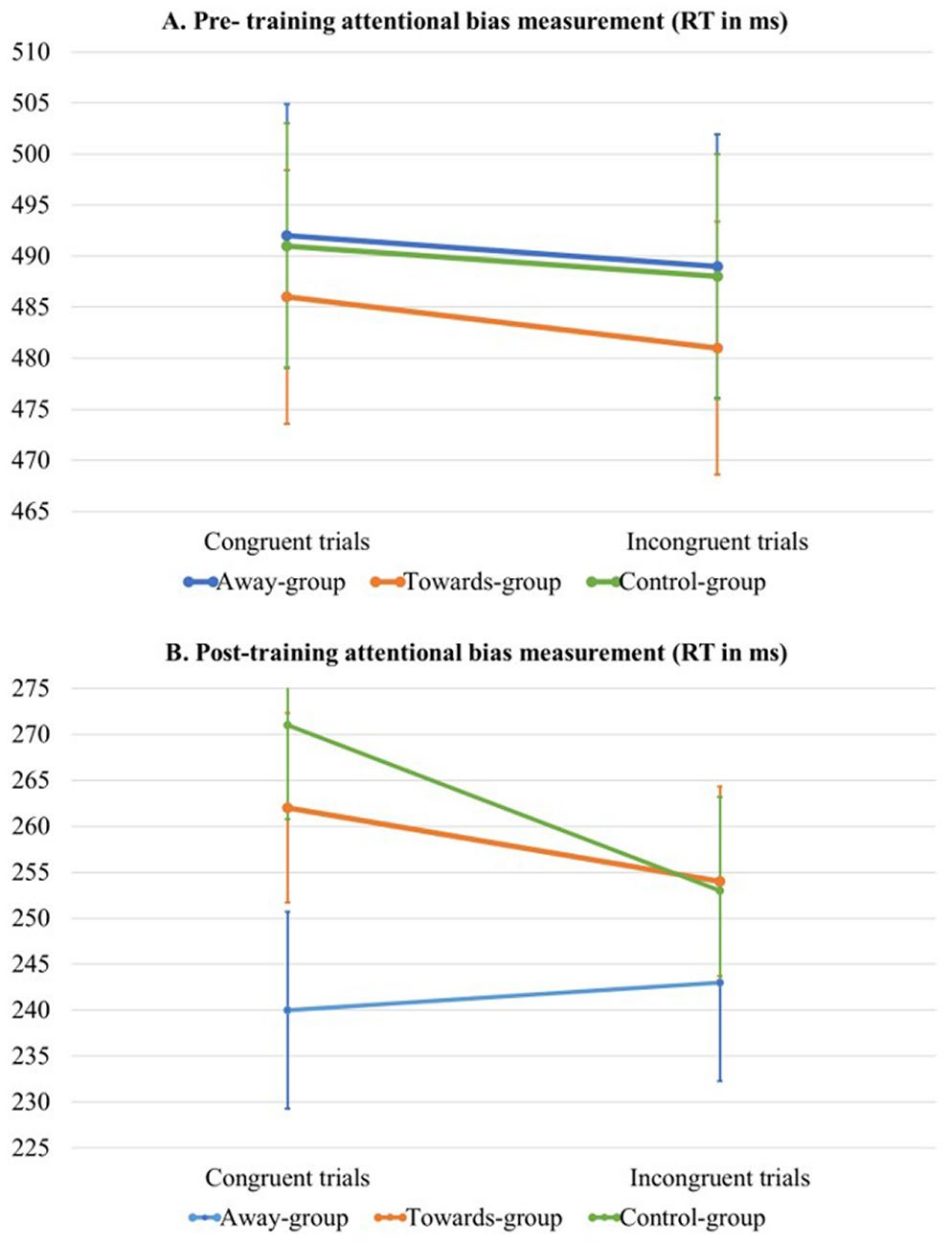
Estimated marginal means per trial type (congruent vs. incongruent) and group (away- vs. towards- vs. control-group) during the pre-training **(A)** attentional bias measurement and the post-training **(B)** attentional bias measurement.

Mixed model analyses of the pre-training attentional bias measurement showed no significant effect of congruency, group or congruency by group interaction, see Model 1 in Table 2A and Figure 2A for visualisation of the data. Therefore, there was no significant attentional bias towards itch in the three groups.

**Table 2:** Mixed-model analyses of the pre-training attentional bias measurement: estimates of the effect of the predictors in the outcome (*ES, in ms*) with standard errors (*SE*), significance level (*p*-value) and 95% confidence intervals of the estimates (95% *CI*) (*n* = 114).

		ES	SE	*p*-value	95% CI
Model 1	(Intercept)	478.60	18.62	<0.001	[442.14; 515.07]
	Accuracy	21.69	3.40	<0.001	[14.26; 29.12]
	Congruency	−4.15	4.68	0.375	[−13.33; 5.02]
	Group	0.11	8.62	0.990	[−16.78; 17.00]
	Group × congruency	0.22	2.22	0.922	[−4.13; 4.57]
Model 2	(Intercept)	513.10	42.77	<0.001	[431.55; 594.66]
	Accuracy	21.71	3.79	<0.001	[14.30; 29.16]
	Congruency	−11.34	6.22	0.069	[−23.53; 0.86]
	Group	0.07	8.32	0.993	[−15.79; 15.94]
	Flanker index	−0.73	0.25	0.005	[−1.210; −0.25]
	Switch cost	0.14	6.88	0.028	[0.020; 0.27]
	Diseng-I	−11.14	6.88	0.108	[−24.24; 1.97]
	PVAQ-I	0.001	0.97	0.999	[−1.14; 1.14]
	PCS-I	1.75	1.19	0.146	[−0.52; 4.02]
	ECIP-I	−1.58	0.96	0.104	[−3.41; 0.26]
	Group × congruency	0.10	1.23	0.966	[−4.28; 4.47]
	Flanker index × congruency	0.15	0.07	0.032	[0.01; 0.28]
	Switching costs × congruency	0.01	0.02	0.768	[−0.03; 0.04]

Model fit statistics; Model 1: AIC = 226,245; Model 2: AIC = 226,233.

After adding the Flanker index, switching costs and self-report questionnaires as covariates (Model 2), results show a significant effect of Flanker index and switching costs on RT during the pre-training block, as well as a significant interaction between Flanker index and congruency. This means that overall RT during the attentional bias measurement was influenced by participants’ attentional inhibition (Flanker index) and their cognitive flexibility (switching costs). More attentional inhibition led to overall faster RT and more switching costs led to overall slower RT. Moreover, the effect of congruency (congruent vs. incongruent) interacted with someone’s ability to inhibit irrelevant information (Flanker index). Specifically, participants with a higher Flanker index showed slower RT during incongruent trials compared to congruent trials during the attentional bias measurement, see Table 2.

Pre-training itch sensitivity AUC scores did not differ significantly between groups before the training, *p*_boot_ = 0.609.

#### 3.2.1. Post-training

Post-training attentional bias measurement data was filtered based on trials with RT < 150 ms, extreme values for the switching costs (*n* = 2), and due to very low accuracy (<0.70) during the training block (*n* = 1). This resulted in a data loss of 16.9%. Again, reliability analyses showed a high mean Spearman-Brown coefficient for congruent trials (0.94; IQR = 0.93; 0.95) and incongruent trials (0.92; IQR = 0.91; 0.93), but the mean Spearman-Brown coefficient for the AB index was lower (0.70; IQR = 0.65; 0.75), indicating lower reliability.

For the post-training measurement of attentional bias, mixed model analyses revealed a significant main effect of congruency, in which RT on incongruent trials was lower compared to congruent trials. This could be interpreted as an attentional bias away from itch stimuli. The analyses also revealed a significant difference between groups. Pairwise comparisons for the main effect of group showed no significant results (all *p* > 0.05). Even though this seems counterintuitive based on the main effect, this can happen because the main effect takes into account all possible comparisons. However, only the pairwise comparisons relevant to the hypotheses were inspected and appeared to be not significant. Furthermore, we found a significant association between pre-training AB-index and RT. This means that a higher AB-index during the pre-training is associated with slightly higher RT during the overall RT during the post-training. Lastly, there was a significant group by congruency interaction effect, see Model 3 in Table 3 and Figure 2B for visualisation of the data. Pairwise comparisons showed a significant effect for congruency in the control group only (*p* = 0.028), with slower RTs for incongruent trials [Estimated Marginal Mean (EMM) = 253.0] compared to congruent trials (EMM = 271.0). Therefore, it can be concluded that the interaction effect between congruency and group is driven by this single comparison within the control group.

**Table 3:** Mixed-model analyses of the post-training attentional bias measurement: estimates of the effects of the predictors on the outcome (*ES in ms*) with standard errors (*SE*), significance level (*p*-value) and 95% confidence intervals of the estimates (95% *CI*) (*n* = 114).

		ES	SE	*p*-value	95% CI
Model 3	(Intercept)	226.68	14.99	<0.001	[197.44; 256.01]
	Accuracy	120.87	6.42	<0.001	[108.09; 133.51]
	Congruency	−28.97	8.86	0.001	[−46.24; −11.51]
	Group	−15.60	5.90	0.009	[−27.13; −4.08]
	Pre – AB index	0.52	0.24	0.032	[0.05; 0.99]
	Group × congruency	10.73	2.07	<0.001	[6.67; 14.79]
Model 4	(Intercept)	205.60	32.17	<0.001	[144.50; 266.82]
	Accuracy	120.90	6.42	<0.001	[108.16; 133.57]
	Congruency	−36.38	9.70	<0.001	[−55.38; −17.37]
	Group	−14.78	6.03	0.016	[−26.22; −3.33]
	Pre – AB index	0.42	0.24	0.089	[−0.04; 0.87]
	Flanker index	−0.23	0.18	0.212	[−0.58; 0.12]
	Switch cost	0.04	0.05	0.383	[−0.05; 0.13]
	Diseng-I	0.45	4.94	0.927	[−8.92; 9.83]
	PVAQ-I	−0.09	0.42	0.919	[−0.89; 0.70]
	PCS-I	1.29	0.86	0.137	[−0.34; 2.91]
	ECIP-I	−0.23	0.69	0.738	[−1.54; 1.08]
	Group × congruency	11.98	2.11	<0.001	[7.85; 16.10]
	Flanker index × congruency	−0.04	0.06	0.559	[−0.16; 0.09]
	Switching costs × congruency	0.05	0.02	0.001	[0.02; 0.09]

Model fit statistics; Model 3: AIC = 195,003; Model 4: AIC = 194,996.

Model 4, with Flanker index, switching costs and self-report questionnaires as covariates (see Table 3), shows significant main effects for congruency and group, as well as a significant interaction effect for group by congruency and a significant interaction effect for congruency by switching costs. This means that after controlling for all these covariates, it can be seen that congruent trials are significantly slower than incongruent trials, which is interpreted as an attentional bias away from itch for all participants. Pairwise comparisons to investigate the main effect of group did not yield significant differences (all *p* > 0.05), but pairwise comparisons of the interaction effect of congruency by group, showed a significant congruency effect for the control group (*p* = 0.017). Lastly, the significant interaction effect between switching costs and congruency showed that higher switching costs, which means less cognitive flexibility, are related to slightly slower RT on incongruent trials. However, the estimate is too low to be interpreted as a meaningful effect (ES = 0.05 ms).

Lastly, itch sensitivity AUC scores post-training did not differ significantly between groups, while controlling for pre-training AUC scores, *p*_boot_ = 0.412.

## 4. Discussion

Results of this study indicated that healthy individuals did not show an attentional bias (AB) towards visual itch-related stimuli. Next, it was found that a single-session attentional bias modification training (ABM) could influence attention towards visual itch-related stimuli in healthy individuals. Across all training groups, participants showed an AB away from itch after the training, i.e., avoidance of itch. However, when looking into the AB effect for specific groups, i.e., the interaction between group and AB, only the sham- training (control) group showed avoidance of visual itch-related stimuli after the training while there was no effect in the experimental groups. Finally, and in contrast with our hypotheses, the ABM-training did not impact upon itch-sensitivity.

While we indeed found an effect of ABM-training on attention to itch, this effect was not as intended, because the experimental groups that were either trained towards or away from itch showed no significant effect. Therefore, we cannot conclude that the ABM-training worked as we assume. This is in line with the most recent findings on ABM-training for itch (16) and also pain (39, 40), as well as the limited findings on preconscious ABM-training for threat (27). In addition to the fact that the current ABM-training had not the expected effect on the AB assessment measures, it also did not show effects on itch sensitivity, although this appeared to be more promising according to earlier findings in pain (17–19). Lastly, the current findings also add to the mixed findings on baseline AB towards visual itch-related stimuli in healthy individuals (9–11, 13). The absence of an AB towards itch at baseline might therefore explain why we did not find specific effects of the current training. Patients with chronic itch, in line with previous research showing a small AB towards pain in patients with chronic pain (41, 42), are expected to display a baseline attentional bias. For patients with chronic itch, the experience of itch is highly relevant and acting upon this experience is probably a relevant goal for patients. However, current ABM trainings in patients with chronic pain are thus far also not very successful (17–19), so it remains unknown how patients with chronic itch would respond to an ABM training for itch.

Recent developments in the field of pain have suggested that AB might be more dynamic, i.e., changes from moment to moment, than current AB assessment paradigms can capture and this might explain why attention bias modification training effects are often not found (43–45). In light of this, we might miss other, probably interrelated, aspects of cognitive bias, such as interpretation and memory biases towards itch (46). Especially interpretation of stimuli might be highly important, because at this moment, we are unaware of the specific interpretation that individuals give to used stimulus materials. To our knowledge only one study asked participants to rate the stimulus material that was used during AB assessment which actually showed that material was not rated very high on its intended dimension (i.e., itchiness or painful in this study) and results indeed showed no AB towards itch or pain in heathy individuals (10). Because the same stimulus material was used in the current study, this might also be true for the current study. In addition, especially for healthy individuals like in the current study, the ABM paradigm lacks personal significance because it is not related to an individual’s goal to relieve an itch. Although participants received an itchy stimulus before the ABM-training, the actual experience of itch had already vanished during ABM, as intended in our case. It is assumed that AB in its original evolutionary function informs us about potential harm to our bodies and to induce adaptive behaviours, but this was not the case in the current study. The idea that individuals only show AB towards itch while experiencing itch is supported by the recent finding that only participants who received a histamine-induced itch stimulus on their skin, showed avoidance of itch-representing stimuli (47). Although the itch-stimulus was not even goal-related in this study, it might at least set a context that was related to itch and hence, increase personal relevance.

The finding that the control-group in the current study actually showed avoidance after the sham-training is surprising. For this group, the training did not differ to the pre-training and post-training assessment, which would not suggest any changes during the post-training. There are no clear explanations for this, but one could speculate about an effect of prolonged exposure and learning which might enhance attentional control, and therefore distraction by the pictures from the actual task. Still, these same effects would have been true for the experimental groups. Interestingly, the current result in the control group is in line with a recent study on preconscious AB towards itch which also showed avoidance in healthy individuals (13). This would suggest that this effect is not yet visible with less exposure and an extensive number of trials is needed to evoke avoidance of itch-related stimuli (13). In the current study, the control-group actually did one long AB assessment without any manipulations which in this sense is comparable to regular AB assessments, in line with earlier findings of preconscious avoidance (13).

In conclusion, the current study suggests that common ABM-training paradigms for itch are not working for healthy individuals as we assume. Development of theories on how cognitive biases in itch, and more specifically attentional biases, work are needed and these should guide the development of new paradigms and research designs. In a second step, the possibility to modify these biases can be investigated, because as long as we do not know how these biases operate we do not know where, when and how we should intervene. This is of course even more important if we consider bias modification trainings in the clinical context where patients with chronic itch are included. All in all, assessment of AB and application of ABM trainings in the clinical setting needs to be investigated in more detail, e.g., by taking the dynamics and context relevant to the individual into account, in the future before any conclusions can be drawn.

## Data availability statement

The raw data supporting the conclusions of this article will be made available by the authors, without undue reservation.

## Ethics statement

The studies involving human participants were reviewed and approved by Psychology Research Ethics Committee, Leiden University. The patients/participants provided their written informed consent to participate in this study.

## Author contributions

AL, JB, DR, SD, GC, and RW designed the study. JB led the data acquisition with substantial support by YS, wrote the initial draft of the manuscript, with help of YS, and revised the manuscript according to the critical feedback of AL, DR, RR, SD, GC, YS, and RW. JB and RR analyzed the data. All authors contributed to the article and approved the submitted version.

## Funding

This research is supported by a Leiden University Fonds project grant (CWB 7510/21-03-2O17/dDM) to AL and an Innovative Scheme (Veni) grant (451-15-019) of the Netherlands Organization for Scientific Research (NWO), granted to AL.

## Conflict of interest

The authors declare that the research was conducted in the absence of any commercial or financial relationships that could be construed as a potential conflict of interest.

## Publisher’s note

All claims expressed in this article are solely those of the authors and do not necessarily represent those of their affiliated organizations, or those of the publisher, the editors and the reviewers. Any product that may be evaluated in this article, or claim that may be made by its manufacturer, is not guaranteed or endorsed by the publisher.
